# Metabolic Optimisation of Regulatory T Cells in Transplantation

**DOI:** 10.3389/fimmu.2020.02005

**Published:** 2020-09-02

**Authors:** Mo Atif, Audrey Mohr, Filomena Conti, Olivier Scatton, Guy Gorochov, Makoto Miyara

**Affiliations:** ^1^Inserm U1135, Centre d’Immunologie et des Maladies Infectieuses (CIMI-Paris), Hôpital Pitié-Salpêtrière, AP-HP, Sorbonne Université, Paris, France; ^2^Unité de Transplantation Hépatique, Hôpital Pitié-Salpêtrière, AP-HP, Paris, France; ^3^Centre for Liver and Gastrointestinal Research, NIHR Birmingham Biomedical Research Centre, University of Birmingham, Birmingham, United Kingdom

**Keywords:** regulatory T cells, Treg, transplant, cell therapy, metabolism, metabolic, mTOR, hypoxia

## Abstract

Regulatory T (Treg) cells expressing the FOXP3 transcription factor are presently under investigation by many teams globally as a cellular therapy to induce tolerance in transplantation. This is primarily due to their immunosuppressive and homeostatic functions. Depending on the type of allograft, Treg cells will need to infiltrate and function in metabolically diverse microenvironments. This means that any resident and circulating Treg cells need to differentially adapt to counter acute or chronic allograft rejection. However, the links between Treg cell metabolism and function are still not entirely delineated. Current data suggest that Treg cells and their effector counterparts have different metabolite dependencies and metabolic programs. These properties could be exploited to optimize intragraft Treg cell function. In this review, we discuss the current paradigms regarding Treg cell metabolism and outline critical intracellular axes that link metabolism and function. Finally, we discuss how this knowledge could be clinically translated for the benefit of transplant patients.

## Introduction

Novel immunomodulatory approaches are required to induce tolerance in solid organ transplantation (SOT) (1, 2). Although spontaneous tolerance has been reported in certain long-term patients (especially post-liver transplant), the majority continue to require ongoing immunosuppression (3, 4). These immunosuppressants have numerous side effects and do not overcome the challenges of delayed allograft dysfunction as well as infectious/neoplastic complications. Hence, there is an urgent clinical need for novel immunomodulatory strategies.

**FIGURE 1:**
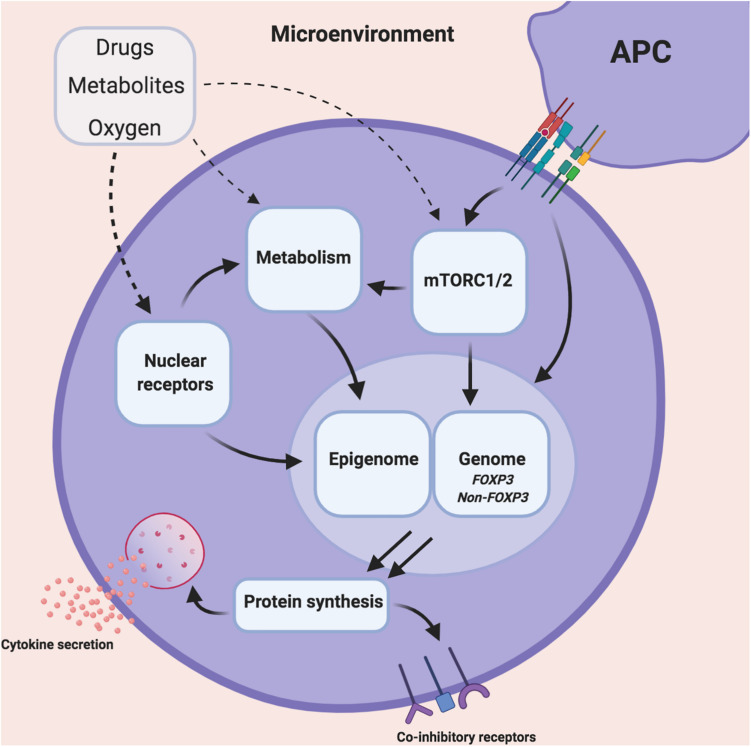
Illustrating the influence of the microenvironment on Treg cell metabolism, epigenome and function. The inflamed tissue microenvironment consists varying concentrations of dietary metabolites, oxygen as well as concomitant immunosuppressants. These entities can individually or collectively modulate various intracellular Treg cell pathways such as mammalian transporter of rapamycin (mTOR), mitochondrial/non-mitochondrial metabolism, nuclear receptors, the epigenome and genome. This modulation has downstream consequences for the Treg cell transcriptome and overall cell function.

**FIGURE 2:**
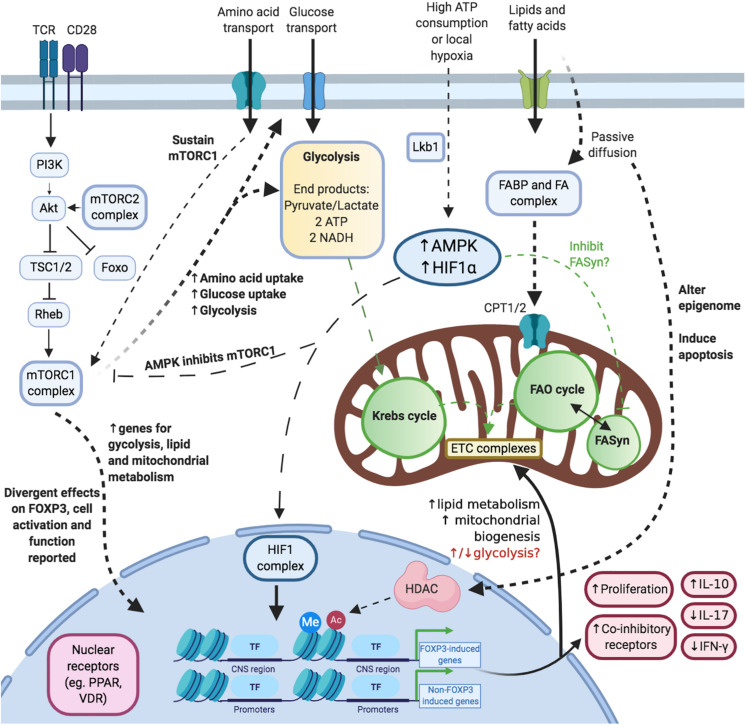
Illustrating the complex and multiple links between Treg cell metabolism, function and the epigenome. **Activation and mTOR:** Upon acute activation, the mTORC1 complex is activated. This process is also promoted (and maintained) via amino acids. **Glucose:** Treg cells upregulate glycolysis and glucose uptake upon acute activation. **Lipids/Fatty acids:** Short- and medium-chained fatty acids are taken up into cells via passive diffusion whereas long-chain fatty acids depend on transporters. Fatty acids affect cellular survival, metabolism and epigenetics. **Hypoxia:** Hypoxia is associated with increased levels of adenosine monophosphate (AMP) and HIF1 complex. These modulate mTORC1 activity, fatty acid synthesis and the transcriptome. **Nuclear receptors:** Nuclear receptors are a key link between the steroidal and non-steroidal ligands and DNA modulation.

Regulatory T (Treg) cells are a CD4^+^ T-cell subset that was first identified as having immunosuppressive effects in mice (5). In SOT, the presence of Treg cells in the periphery and the graft has been associated with allograft tolerance (6–8). These cells perform their functions through a range of effector cell contact-dependent and –independent mechanisms (9–13). However, in recent years several groups have identified that these functions are tightly linked to Treg cell metabolism and epigenome too (14–16) (Figure 1). These are important links to delineate as Treg cells need to survive and function in the metabolically demanding microenvironment of a chronically inflamed allograft. Moreover, novel data demonstrates that metabolites such as acetyl coenzyme A (acetyl CoA) and fatty acids do not just partake in different metabolic programs but can directly modulate the epigenome too (17, 18) (Figure 2). Through either DNA acetylation or DNA/histone methylation, these metabolites facilitate a complex network involving the epigenome, metabolism, and function of Treg cells. Delineating this network is important to understand Treg cell behavior in the allograft. In this review, we discuss Treg cell metabolism and interlink it with their diverse functions.

From a clinical perspective, the initial data from Phase I trials in transplant and non-transplant settings has shown Treg cells to be safe (1, 19–22). The current Good Manufacturing Practice (GMP) protocols center on the hypothesis that the infusion of expanded autologous Treg cells could modulate the inflamed allograft microenvironment in favor of immunoregulation instead. In parallel, Treg cells are also being modified in different ways to augment their potential activity e.g., expansion period, antigen-specificity, pharmacological agents (1, 23–25). However, metabolic modulation of Treg cells in this context has not been widely performed (26). In this review, we discuss how metabolic pathways can be exploited to improve the efficacy of Treg cell therapies in transplantation.

## Linking Treg Cell Functions and Metabolism

Despite the heterogeneous phenotype of Treg cells, the expression of the Forkhead Box Protein 3 (FOXP3) transcription factor (TF) is considered as a reliable indicator of Treg cells (1, 9, 27, 28). FOXP3 in combination with T-cell receptor (TCR) activation, IL-2, mammalian transporter of rapamycin (mTOR) complexes, and others play a key role in promoting Treg cell proliferation and function.

However, to sustain these metabolically demanding processes, Treg cells rely on various stimuli, metabolites, and metabolic pathways (14, 15, 25) (Figure 1). These stimuli are dynamic (e.g., oxygen gradient, glucose/lipid availability) and vary depending on the type of organ and disease (29–31). In recent years, numerous publications have demonstrated how the manipulation of these metabolic factors can in turn modulate Treg cell function. This is important as Treg cells (circulating or resident) need to survive and function in diverse microenvironments (32, 33). During inflammatory diseases e.g., allograft rejection), the microenvironment is infiltrated by other effector cells, who will also start competing for metabolites to survive and function (34). From the perspective of SOT, the aim is that by exploiting cellular metabolism, one could augment Treg cell survival and function in the inflamed allograft microenvironment (Figure 1). This would be an additional strategy to support either tissue-resident Treg cells or infiltrating Treg cells as part of a cellular therapy protocol.

As we discuss in the following sections, many studies into Treg cell immunometabolism have identified drugs/metabolites that mediate their roles through key intracellular axes. As these axes interlink both Treg cell metabolism and function, their modulation is a novel approach with the potential for clinical translation in SOT. In the following sections, we discuss Treg cell metabolism in-depth and contextualize this through the following three intracellular axes (Figure 1):

1. mTOR.
2. Hypoxia.
3. Nuclear Receptors.

For clarity, in this review we will describe naturally occurring Treg cells as thymus-derived Treg (tTreg) cells, the induced Treg cells as iTreg cells and peripheral Treg cells as pTreg cells (35).

### Glycolysis in Treg Cells

Upon activation via co-stimulation of the T-cell receptor (TCR) and CD28, the signaling cascades promote glucose uptake (via Glut1 transporters) and glycolysis (Warburg effect) instead (36, 37) (Figure 2). This process occurs in the cytoplasm and generates 2 units of adenosine triphosphate (ATP) per mole of glucose converted to CO_2_ (38). In parallel, Treg cells increase FOXP3 expression, cellular proliferation, and immunosuppressive functions (14, 15, 37, 39). It is not yet established why Treg cells switch to this less efficient ATP-generating metabolic program rather than continuing with oxidative phosphorylation (OXPHOS) – especially as cellular activation increases metabolic demands in terms of protein synthesis.

A further process to comprehend is the regulation of the end products of glycolysis (either pyruvate or lactate) (40). The balance between both of these products depends on the activity of lactate dehydrogenase (LDH) as well as the levels of nicotinamide adenine dinucleotide (reduced form; NADH and oxidized form; NAD^+^) (41). This is relevant to Treg cells because FOXP3 can modulate LDH to prevent lactate formation and form pyruvate instead (40). Moreover, in a high lactate low glucose environment, Treg cells can convert lactate to pyruvate too. Whilst lactate may negatively impact on T-cell proliferation as a whole, it does not impact Treg cell immunosuppression. This is of particular relevance to tumoral microenvironments which are known to have high levels of local lactate and Treg cell accumulation (40).

If not converted to lactate, the resulting pyruvate is transported into mitochondria to be converted via pyruvate dehydrogenase into acetyl-CoA and NADH (42). This acetyl-CoA molecule subsequently enters the Krebs cycle (42).

### Fatty Acid Metabolism in Treg Cells

In addition to glycolysis, Treg cells rely on lipid metabolism to meet their metabolic requirements. In the murine tumor setting, Treg cells were shown to express both genes for glycolysis and as well as the pentose phosphate pathway (PPP) (16). The end products of this pathway could be used for fatty acid synthesis (FASyn) or protein synthesis. Tumoral Treg cells also stored lipids intracellularly and preserved the ability to perform fatty acid oxidation (FAO) too. Overall, this data demonstrated that murine tumoral Tregs were capable of glycolysis and OXPHOS mediated via FASyn/FAO.

However, it is unclear why Treg cells should maintain the FASyn and FAO programs as both would theoretically at least nullify the effects of the other (Figure 2). Indeed, this question has been studied by a few teams in recent years (36, 43, 44). In one study involving murine T-cells, the inhibition of acetyl-CoA carboxylase (key enzyme for FASyn) in naïve CD4^+^ T-cells, via either genetic knockout (KO) or pharmacological means, diverted the differentiation process toward FOXP3^+^ cells instead of IL-17A-producing cells (44). These FASyn-inhibited iTregs were just as immunosuppressive *in vitro* as control iTregs. Moreover, the control iTregs and FASyn-inhibited iTregs had similarly reduced levels of genes for glycolysis and glutaminolysis. Both took up equal amounts of palmitate too. Put together, modulating fatty acid metabolic pathways could be a strategy to polarize iTreg cell differentiation and function.

A further yet important line of inquiry is regarding how FOXP3 can modulate lipid metabolism (Figure 2). FOXP3^+^ tissue Treg cells take up long-chain fatty acids (lcFAs) into via the CD36 receptor (45). However, short and medium-chained fatty acids (scFAs and mcFAs, respectively) diffuse passively across the cytoplasm and mitochondrial outer/inner membranes to participate in FAO (46). In a series of eloquent experiments using a murine lymphoma cell line (EL4), Howie D. et al. demonstrated the effects of FOXP3 on lcFAs metabolism (39). They transfected EL4 cells with a FOXP3-ERT2 construct such that the administration of an estrogen modulator (4-HT) would translocate this construct to the nucleus. These transfected FOXP3^+^ cells had an increased oxygen consumption rate (OCR) at baseline than the non-transfected controls. The OCR was further increased after being cultured with palmitate (long-chain fatty acid, C16). Interestingly, in EL4-FOXP3 cultures without palmitate, the addition of etomoxir reduced OCR rates. This demonstrated that part of the increased FOXP3-mediated OXPHOS was due to the FAO of endogenous fatty acids. These cells in parallel also increased the expression of genes for mitochondrial electron transport chain (ETC) complexes. A similar effect was demonstrated in 24 h activated human Treg cells (CD4^+^CD25^+^FOXP3^+^) as they too augmented genes specific for mitochondria. This further confirmed the role of FOXP3 in promoting mitochondrial-based metabolism. The same group also studied whether FOXP3 could promote Treg cell survival in a high-fat microenvironment. They found that murine Treg cells were less apoptotic after 18 h of cultures with lcFAs compared to Teff cells. This was an interesting observation as they found that Treg cells took up more fluorescent-palmitate. This indicated that FOXP3 could indeed be inhibiting the apoptosis-inducing effects of palmitate. In their EL4-FOXP3 cells, they identified the mechanism for this effect as being due to increased FAO of palmitate. Collectively, all these data demonstrate how FOXP3 promotes OXPHOS through increasing FAO of lcFAs and mitochondrial ETS complex synthesis.

However, before Treg cells can engage lcFAs in FAO, the lcFAs need to be transported across the cytoplasm and enter the mitochondria (Figure 2). These two processes are facilitated by the fatty acid-binding proteins (FABP) and the carnitine palmitoyltransferase transporters (CPT1/2), respectively (47). Treg cells predominantly express the FABP5 transporter although other isoforms have been described (48, 49). Recent work by Field C. et al. demonstrated that pharmacological inhibition of FABP5 in newly differentiated iTregs switched their metabolic program from OXPHOS to glycolysis (as evidence by the extracellular acidification rates; ECAR) (48). These cells also developed an altered mitochondrial structure and synthesized fewer proteins specific for the mitochondrial ETCs. As a consequence, lcFAs were unable to engage in FAO and the Krebs cycle. However, in an interesting demonstration of the roles of lcFA metabolism in modulating Treg cell function, they also identified that FABP5 inhibition in iTregs and human Treg cells led to increased *in vitro* suppression via IL-10 secretion. The mechanism for this effect involved the release of mitochondrial DNA and subsequent increase in interferon signaling via the innate pattern recognition pathway, cycle GMP-AMP synthase (cGAS) and Stimulator of Interferon Genes (STING). Collectively, these data suggest that inhibiting lcFA-FAO metabolic pathway may be more favorable as an approach to increasing Treg cell suppressive function. They also suggest that the overall effects of FAO on Treg cells are broader than just supplementing the Krebs cycle. It is plausible that various intermediates produced during FAO such as acetyl-CoA and reduced flavin/nicotinamide adenine dinucleotides (FADH/NADH) could be interfering with Treg cell function through yet unknown mechanisms.

The actual FAO process occurs in the mitochondria and involves the formation of one acetyl-CoA molecule per cycle (50). The acylated fatty acids keep entering the FAO cycle until a 2-carbon unit can no longer be formed. Each cycle also produces an NADH and FADH_2_ molecule that donate additional electrons to the ETCs (50). With regards to producing ATP, this is a very efficient process as the full metabolism of a 16-chain fatty acid (palmitate) leads to 106 molecules of ATP – much more than via glycolysis or glucose substrate-only OXPHOS (50). This may explain why in a glucose-deprived tumoral microenvironment, Treg cells utilize CD36 to maximize fat uptake as a fuel to meet their metabolic demands (45).

### Krebs Cycle and Mitochondrial Complexes in Treg Cells

The purpose of the above pathways is to generate enough acetyl-CoA to feed into the Krebs cycle and then generate sufficient ATP through the mitochondrial ETC. This is an important process in Treg cells as links between FOXP3, ETC synthesis, and cellular functions have been described (39, 51, 52) (Figure 2). Although the mechanism was not uncovered, the induction of FOXP3 in iTreg cells correlated with increased expression of mitochondria-associated genes (39). Moreover, a recent manuscript involving mice demonstrated that complex III *per se* was key to promoting Treg cell suppressive function (40, 52). The Treg-specific knockout of complex III was associated with reduced immunosuppressive capacity and increased DNA methylation status – without affecting FOXP3 expression, cell frequency, or co-inhibitor receptor expression (52). These mice also developed a general inflammatory condition (similar to that of *scurfy* mice) and did not live beyond 4 weeks of life. Put together, these data identify the additional role of mitochondrial metabolism alongside FOXP3 in facilitating Treg cell function.

### Do tTreg and iTreg Cells Have Different Metabolic Programs?

Although the majority of FOXP3 + Treg cells are of thymic-origin, a small proportion are induced (iTreg cells *in vitro*) from effector T (Teff) cells via exposure to different cytokines in the microenvironment (35). Through studying the conversion of effector T (Teff) cells to iTreg cells, one can identify how their metabolic phenotype changes via *de novo* FOXP3 induction. This is not possible with tTreg cells which already express FOXP3 upon entering the peripheral circulation.

*Ex vivo* non-activated murine tTreg cells (defined as CD4^+^FOXP3^+^) have a higher baseline proliferative status (Ki67) and express more Glut1 (glucose transporter) than Teff cells (37). Just as FOXP3 expression is a Treg cell-lineage identifier, there is increasing data that OXPHOS is their key lineage metabolic program (37) (Figure 2). This supported by metabolomics data of resting human tTregs (defined as CD4^+^CD25^+^ or CD4^+^CD127^lo^CD49b^–^ in the referenced study), which found Treg cells to produce increased glycolysis- and OXPHOS-related metabolites such as lactate, α-ketoglutarate, and succinate in comparison to Teff cells or naïve CD4^+^ T-cells (53). In comparison, another study utilizing a proteomics approach, demonstrate slightly different results (54). Although resting human Treg cells (CD4^+^CD25^+^CD127^lo^) did indeed express a greater quantity of glycolysis-related proteins than Teff cells (CD4^+^CD25^–^), the Teff cells expressed a greater quantity of proteins related to the Krebs cycle and the mitochondrial ETC instead. Moreover, these proteomic differences did not translate into differing metabolic programs as the Treg cells consistently had a higher baseline rate of ECAR and OCR. Collectively, these datasets suggest that both glycolysis and oxidative phosphorylation are a fundamental part of the baseline tTreg cell metabolic program.

In comparison, acutely activated human tTreg cells in the *in vitro* setting initially reduce their rate of ECAR and OCR in comparison to Teff cells (43). However, after a ∼1-week stimulation with anti-CD3 antibody, IL-2, and antigen-presenting cells (APCs), these Treg cells had significantly higher rates of ECAR and OCR than corresponding Teff cells. The activated Treg cells also took up more of the fluorescent glucose dye than Teff cells despite having similar levels of Glut1 expression. Moreover, the importance of FAO was demonstrated in these Treg cells too as the addition of palmitate to cultures further increased the OCR. Put together, these data suggested that tTreg cells were better equipped than Teff cells to meet their additional metabolic requirements through upregulating both glycolysis and FAO upon acute activation (Figure 2).

From a functional perspective, the inhibition of either glycolysis or FAO was found not to profoundly affect Treg cell immunosuppressive capacity (54). However, the data from this study contrasts with data from other published work that reported Treg cell immunosuppressive capacity to be more significantly reduced when glycolysis, FAO, or lipid/cholesterol synthesis were individually inhibited (53). When reconciling these divergent results, we noted key differences in the experimental design of the suppression assay e.g., responder cell type, the dose of metabolism inhibitors, pre-culture period, and the readout dye (thymidine/carboxyfluorescein). It is important to take these differences into account when considering the evidence base. Collectively, these data demonstrate that the link between tTreg cell metabolism and immunosuppressive function is not fully delineated.

With respect to iTreg cells, the increase in the rate of glycolysis in murine iTreg cells was less pronounced compared to *de novo* induced Th1/2/17 cells (36). These iTregs also expressed less Glut1. However, similarly to the tTreg cells from the above-discussed studies, these iTregs demonstrated dependence on lipid metabolism too. They oxidized significantly more palmitate than their non-Treg counterparts. Moreover, when the FAO inhibitor, etomoxir, was added to the culture system (albeit at a relatively high dose), both the oxidation was inhibited as well as the upregulation of FOXP3. This effect was also identified by another team studying murine iTregs as they demonstrated an association with FOXP3 upregulation and increased OXPHOS rates (40).

All in all, these data suggest that tTreg and iTreg cells depend on both glycolysis and FAO to meet their metabolic demands. These processes are upregulated during activation and their inhibition affects Treg cell proliferation and function (Figure 2).

### Explaining the Variation in Published Experimental Literature

**FIGURE 3:**
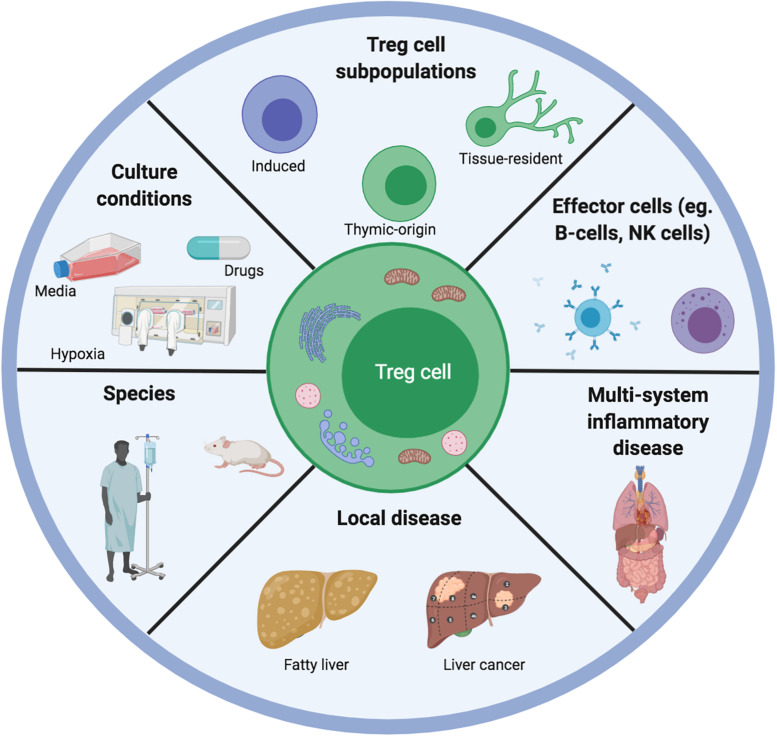
Illustrating the range of variables that could explain differences between published data on Treg cell metabolism and function. Different teams work on Treg cells from different species, different tissue systems and disease settings. This has implications for the type of microenvironment that the Treg cells will have been adapted for. Moreover, any subsequent *in vitro* or *ex vivo* experiments are often carried out in different culture conditions involving different types of media, concomitant pharmacological agents as well as atmospheric oxygen concentrations. Furthermore, the competition for metabolites is different depending on the presence of competing effector cells as well as the constitutive Treg cell metabolic program.

When considering the relevant literature on Treg cell metabolism, it is important to take into account a range of factors (Figure 3):

• Culture conditions (*in vivo/ex vivo/in vitro*).
• Treg cell subtype (thymic, peripheral, induced, FOXP3^+^, and CD25^+^CD127^lo^).
• Disease (graft rejection, tumor, and autoimmunity) or healthy tissue.
• Species (murine and human).
• Microenvironment (competing cells, substrates, oxygen gradient, and drugs).

As is evident from our discussions above, a challenge with the current *in vitro* metabolic assays is ensuring that they reflect *in vivo* physiology. The concentrations of substrates, their competing cells, and the ongoing disease process are constantly evolving when *in vivo*. To study glycolysis, certain studies we discuss in this review have used the inhibitor, 2-deoxyglucose (2-DG) (16, 55). However, there is data demonstrating that this agent can also have off-target effects in terms of triggering kinase pathways e.g., Akt/Erk) (55, 56). Moreover, to study FAO, different studies have used different doses of palmitate (up to 1000 uM) (36, 39). The challenge here is that palmitate is not an exclusive fatty acid in the microenvironment and the concentration can be affected by albumin levels too (36, 57, 58). Finally, certain assays have used etomoxir (mitochondrial lcFA uptake inhibitor) to block CPT1 however, there is data demonstrating that etomoxir acts “independently of CPT1” instead in T-cells (59). A further issue of concern is regarding etomoxir dosing as it has off-target effects above the dose of 5 uM (60). These are only some of the extraneous variables to consider. Overall, our key message is that the relevant data must be contextualized within the limitations of the respective assays.

Having discussed Treg cell metabolism in detail, in the next sections we outline the following key internal axes that connect both metabolism and function in Treg cells (Figure 1):

1. mTOR.
2. Hypoxia.
3. Nuclear Receptors.

## mTOR: The Immunometabolic Highway

The mTOR complexes play a central role in Treg cell metabolism and function (14, 61) (Figure 1). Their ability to sense upstream changes in the microenvironment and subsequently modulate Treg cell metabolism/function that makes them the highway of immunometabolic modulation (61).

mTOR signaling is facilitated through mTOR being linked with other adapter proteins in the form of mTORC1 and mTORC2 complexes (61). The phosphorylation signaling cascade upstream of mTOR starts with stimulation of either the TCR complex or CD28 (62) (Figure 2). This triggers sequential phosphorylation of phosphoinositide 3-kinase (PI3K), phosphoinositide-dependent kinase 1 (PDK1) and then protein kinase B (Akt). Akt subsequently inhibits the heterodimeric tuberous sclerosis complex (TSC1/2) to maintain Rheb protein activity (63). Finally, Rheb can directly and indirectly increase mTORC1 activation (62, 64). This promotes Treg cell immunosuppressive function, prevents the onset of autoimmunity and maintains tissue homeostasis (14).

### How Is mTOR Signaling Modulated?

From a metabolic perspective, mTORC1 activity can be modulated upstream via essential amino acids and hypoxia (*discussed in the next chapter of this review*) (65). Essential amino acids e.g., arginine, leucine, isoleucine) are known as such as they must be acquired from dietary consumption. Although these play a key role in DNA and protein synthesis, they are also vital in promoting Treg-specific mTORC1 activity (15, 66). The evolutionary reasons for this relationship remain unknown.

Amino acids are taken up through dedicated receptors such as SLC7A1, SLC7A5, SLC3A2/CD98, and ASCT2 (15, 66, 67). The expression of these receptors is further increased upon TCR stimulation to optimize amino acid uptake. This is an important mechanism as its inhibition reduces mTORC1 activation (15, 67). Upon acute cellular activation, the ongoing presence of amino acids such as arginine and leucine sustains activation of mTORC1 as well as of the Treg cell itself (via increased *cytotoxic T-cell lymphocyte antigen-4;* CTLA4 and *inducible T-cell costimulator;* ICOS) (15, 68). The inhibition of amino acid uptake receptors has been shown in murine models to reduce *in vivo* Treg cell quantity, cellular proliferation, and suppressive capacity. Moreover, this effect was specific to Treg cells and not Teff cells – thus further confirming a key role for essential amino acids in these cells. Once inside the Treg cell, amino acids activate the Rag small GTPases (such as RagA/B), which alongside the protein Rheb, recruit mTORC1 to lysosomes (15, 68). These are critical processes as murine KO models of either Rag GTPases or Rheb proteins have demonstrated the mice to have Treg cell metabolism/function and they all developed an autoimmune disease similar to that of *scurfy* mice.

### How Does mTORC1 Connect Metabolism and Function?

Resting CD4^+^FOXP3^+^ (Treg cells) have higher levels of constitutive mTORC1 activity than naïve CD4^+^ T-cells or Teff cells (68, 69). Upon activation by anti-CD3, the phosphorylation of S6 and 4E-BP1 (indicators of mTORC1 activity) was increased alongside key functional markers such as CTLA4 (68, 69). These activated Treg cells were also more immunosuppressive.

From a metabolic perspective, resting non-activated Treg cells (isolated either as CD4^+^CD25^+^ or CD4^+^CD127^lo^CD49b^–^) were found to have higher expression levels of genes involved in glucose metabolism e.g., *Glut1, Glut3, PKM2*) and lipid metabolism (*cpt1, fasn, acc1*) than Teff cells (53). Upon Treg cell activation, the increase in mTOR signaling upregulated interferon regulatory factor 4 (IRF4) which further promoted genes for cellular growth, glycolysis, OXPHOS, fatty acid metabolism amongst others (14). Moreover, transfecting tTreg cells with Rheb to upregulate mTOR signaling further increased glucose uptake and glycolysis (53). All this data collectively indicated that promoting mTORC1 activity could also promote Treg cell activation, function, and support both the glycolysis and OXPHOS metabolic pathways.

However, numerous others have demonstrated often divergent effects of mTOR signaling on Treg cells. For example, in a Treg-specific Raptor KO murine model (to inhibit mTORC1 activity), the mice demonstrated an increase in CD4^+^FOXP3^+^ (Treg cells) (69). These Treg cells were still immunosuppressive during *in vitro* assays however they were unable to inhibit colitis development or the *scurfy* phenotype of *in vivo* murine models. Furthermore, from a metabolic perspective, the Raptor KO Treg cells had lower levels of ECAR and OCR. They also downregulated genes for cholesterol and lipid biosynthesis. In particular, cholesterol biosynthesis was demonstrated mechanistically as being important in promoting Treg cell activation, proliferation, and function. Collectively, these data demonstrated that constitutive *in vivo* mTORC1 signaling was important and that mTORC1 played a critical role in promoting lipogenic metabolism and Treg cell function.

These findings were taken further by another group who developed two different murine models to delineate mTORC1 activity; Treg-specific KO of RagA/B GTPases (amino acid sensors) and Treg-specific KO of Rheb1/2 (15). As expected, mTORC1 activity was relatively reduced (not completely inhibited) upon TCR-stimulation in both models compared to wild-type mice. From a functional perspective, the cells demonstrated reduced *in vitro* immunosuppressive capacity – although FOXP3 expression was unaffected (15). Put together, these data suggest that both a combination of mTORC1-related and non-mTORC1-related effects of Rag/Rheb protein signaling could be involved in Treg cell function.

From a metabolic perspective, Treg cells from both models also had reduced rates of glycolysis and oxidative phosphorylation (15). Indeed, the RagA/B KO mice also had fewer mitochondria, reduced mitochondrial function, and superoxide levels. The analysis of the transcriptomes of activated Treg cells of both KO models compared to wild-type Treg cells demonstrated upregulation in pro-inflammatory genes e.g., interferon-gamma, tumor necrosis factor-alpha) as well as a downregulation in genes for cellular proliferation e.g., myc, G2M checkpoint) and oxidative phosphorylation (15). However, within these transcriptomic alterations, there was also a divergence in terms of the effects of metabolism. The RagA/B KO Treg cells upregulated genes involved with lysosomes and lipid metabolism and downregulated those specific for mitochondrial complexes. In comparison, the Rheb1/2 KO Treg cells downregulated genes for metabolizing fatty acids and cholesterol without any changes in mitochondrial biosynthesis/function. Collectively, these data demonstrate that mTORC1 activity plays an important role in Treg cell activation, function, and increased metabolic demands (via glycolysis and OXPHOS). However, they also demonstrate that the RagA/B and Rheb1/2 proteins differentially modulate lipid metabolism and OXPHOS. Exactly how these divergent metabolic pathways are reconciled during Treg cell activation and increased mTORC1 activity is yet unknown.

### How Does mTORC2 Connect Treg Cell Metabolism and Function?

Concerning mTORC2, its role in Treg cells has been relatively less well-defined compared to mTORC1. mTORC2 activates Akt via phosphorylation at the serine residue (position 473) (70) (Figure 2). In response, Akt phosphorylates mTORC1 and the FOXO transcription factors (70). Phosphorylation of the FOXO TFs propagates their subsequent degradation via ubiquitination. Hence, any experiments involving mTORC2 inhibition need to take into account that the downstream effects could involve reduced mTORC1 activity as well as increased FOXO levels (Figure 2).

Studies into the effects of mTORC2 modulation on Treg cells have generally demonstrated mixed effects on their phenotype and function (69–71). In one study, the murine model of Treg-specific mTORC2 KO (Rictor−/−) demonstrated reduced frequencies of Treg cells in all peripheral tissue (except the thymus) (69). However, the phenotype of the Treg cells was unaltered (CTLA4, ICOS levels). They also remained immunosuppressive during *in vitro* assays. From a metabolic perspective, their mitochondrial function was also unaffected.

These findings are in contrast to those of another group who developed murine models with a Treg-specific loss-of-function modification of FOXP3 with and without an additional Rictor KO (mTORC2 inhibition) (71). Through these single- and dual-mutated mice, they were able to delineate the relationship between functional FOXP3^+^ Treg cells and those without a functioning mTORC2 component (71). The phenotypic analysis of Treg cells from the dual-mutated mice demonstrated increased expression of markers such as *glucocorticoid-induced tumor necrosis factor receptor* (GITR) and ICOS. These cells had the capacity to secrete more IL-4, IL-10, and less IFNγ compared to the FOXP3-mutation mice (IL-17A was unchanged). They were also more immunosuppressive during *in vitro* suppression assays compared to Tregs from the FOXP3-mutation mice. However, importantly, the suppressive capacity of the FOXP3-only mutated Treg cells improved with rapamycin pre-treatment. Collectively, these data suggested that mTORC2 inhibition promoted Treg cell activation status, Th2-like differentiation, and immunosuppressive function.

From a metabolic perspective, the Treg cells of the FOXP3-mutation mice upregulated glycolysis and OXPHOS (as demonstrated via increased ECAR and OCR, respectively) (71). This was reflected by upregulation of enzymes and metabolites involved in glycolysis and the Krebs cycle. Furthermore, the inhibition of glycolysis in these cells reduced their secretion of IFNγ, IL-4, and improved their *in vitro* immunosuppressive function. However, this metabolic reprogramming was attenuated in Treg cells from mice with the additional Rictor-KO (mTORC2 inhibition). Put together, these data suggest that FOXP3 and mTORC2 have opposing effects on Treg cell phenotype, metabolism and function (71). However, it is yet unknown how this relationship is affected by other metabolites such as amino acids (which sustain mTORC1 activity) or fatty acids.

## Hypoxia

Understanding the role of hypoxia in Treg cells is especially important with regards to delineating their survival and function in the physiological hypoxia liver (72). During hypoxia or a state of high ATP consumption, there is a proportional increase in intracellular AMP as well as *hypoxia-inducing-factor-1-alpha* (HIF1α) transcription factor (HIF1α) (65). Both of these cofactors utilize different signaling pathways to modulate Treg cell functions.

The proportional increase in AMP leads to adenosine monophosphate kinase (AMPK) phosphorylation and activation by liver kinase B1 (Lkb1). This Lkb1 enzyme is crucial for Treg cell metabolism and function (65). The activated AMPK then inhibits Rheb and phosphorylates Raptor (mTOR adapter protein) to inhibit mTORC1 activity (73). Interestingly, activated AMPK also in parallel, inhibits acetyl-CoA carboxylase (ACC) to prevent fatty acid synthesis (74). Although this latter mechanism has not been demonstrated in Treg cells, it may be a potential metabolic adaptation during a low ATP state to divert available intracellular lipids toward acetyl-CoA-generating FAO instead.

In comparison, HIF1α levels increase during hypoxia as it is unable to be degraded via the proteasome-based mechanism (75). This would normally involve prolyl hydroxylation, subsequent binding to von Hippel-Lindau protein, and ubiquitination (75). Without this degradation, HIF1α forms a complex with its counterpart HIF1β, which then binds to specific hypoxic response elements (HRE) to influence Treg cell metabolism/function (Figure 2). However, the exact role of HIF1α in Treg cells is not clear as the data we discuss below describe contrasting effects.

### FOXP3

Firstly, hypoxia appears to differentially modulate tTreg and iTreg cells. When murine tTreg cells (CD4^+^FOXP3^+^) were cultured for 5 days under acute hypoxia (1% O_2_) with anti-CD3/28-based activation and IL-2, there was no change in their FOXP3 expression (76). This was also matched by *in vivo* data demonstrating that tTreg cells from murine models of CD4-specific KO of HIF1α had comparable levels of FOXP3 to control mice (76, 77). However, when CD4^+^ T-cells from control mice were cultured under acute hypoxia with activation, IL-2 ± TGFβ, there was a proportional and significant increase in cells expressing FOXP3 (76, 78). This increase was also demonstrated *in vivo* in mice exposed to environmental hypoxia (10% O_2_ for 24 h) (76). Put together, these data suggest that hypoxia does not alter FOXP3 expression on tTregs but induces it in non-Treg cells.

### Immunosuppressive Function

In an *in vitro* suppression assay, the hypoxia-induced iTreg cells discussed above were cultured at different ratios with Teff cells (CD4^+^CD25^–^) and anti-CD3/28 antibodies for 72 h. These iTregs were better able to suppress Teff proliferation than their normoxic counterparts (78). In comparison, a rather different result was achieved using Treg cells from murine models of CD4-specific HIF1α KO (76). In this study, CD4^+^CD25^+^ Treg cells were only slightly less immunosuppressive at the higher ratios of Tregs:Teff cells (1:1, 1:2) compared to control Treg cells. However, there were no differences in immunosuppression between the two groups at the lower ratios. Moreover, adoptive transfer of these HIF1α KO Treg cells into a murine model of T-cell-mediated colitis demonstrated that the Treg cells were unable to inhibit weight loss or the development of colitis. Put together, these data suggest that HIF1α also differentially affects the immunosuppressive functions of tTreg/iTreg cells (76).

A further unknown question is how *in vivo* Treg cells function in hypoxic inflammatory microenvironments (79). In one study, involving tTreg cells, their acute activation was associated with an increase in HIF1α was identified compared to normoxic controls (53). The PI3K-mTOR pathway was crucial in upregulating HIF1α (53, 79). In addition, HIF1α played an important role in augmenting tTreg cell function as pre-culturing these cells for 24 h with a HIF1α-inhibitor reduced their ability to suppress naïve T-cell proliferation (53). Put together, these data suggest that tTregs could have augmented HIF1α levels and immunosuppressive function in inflammatory hypoxic microenvironments. This could have positive implications for their utilization in physiologically hypoxic liver allografts.

### Treg Cell Differentiation and Stability

Concerning iTreg cells, an important question is whether hypoxia could influence the differentiation of CD4^+^ T-cells to Th17/iTreg cells. In one study, murine splenocytes were cultured under hypoxia with anti-CD3 antibody, IL-2, TGFβ for 5 days before staining (76). The authors identified an increase in FOXP3 expression amongst the CD4^+^ T-cells cultured under hypoxia as opposed to normoxia. Conversely, the CD4^+^ T-cells did not change their expression levels of RORyt or secretion of IL-17A. Indeed, they found that they had to deliberately culture their splenocytes under Th17-differentiating conditions to induce these changes. This study suggested that HIF1α had an additive effect on differentiation rather than a polarizing toward Th17 or iTregs.

However, a different study using a pure naïve CD4^+^ T-cell population identified that HIF1α was indeed the key factor in influencing differentiation to Th17 cells (80). Under Th17-differentiating conditions, they identified that *signal transducer and activator of transcription 3* (STAT3)-induced augmentation of HIF1α expression promoted transcription of RORyt. Both HIF1α and RORyt then formed a complex with the histone acetyltransferase, p300, to bind to the IL-17A promoter region. In comparison, when naïve CD4^+^ T-cells from mice with CD4^+^-specific HIF1α KO were cultured under Th17-differentiating conditions, they identified an increase in FOXP3 expression compared to wildtype controls. Moreover, when the same cells were cultured under iTreg-differentiating conditions instead, the HIF1α^–/–^ cells expressed much more FOXP3 than wildtype controls. All of this suggested that HIF1α was negatively affecting FOXP3 levels. Indeed, they confirmed this hypothesis by demonstrating that HIF1α utilized the ubiquitin-based degradation mechanism to directly target and degrade FOXP3. Overall, these studies suggest that HIF1α can modulate Th17/iTreg differentiation through epigenetic and metabolic means.

A further question is whether HIF1α can modulate Treg cell stability. This was demonstrated using a Treg-specific model of von Hippel-Lindau (VHL) KO to study the effects of HIF1α overexpression in Treg cells only (81). These mice did not survive beyond 6–11 weeks and had increased Th1 infiltrates in all tissues. Interestingly, the Treg cells from this model had no alterations in their baseline activation or functional phenotypes e.g., CD25, CTLA4, CD69). However, after 48 h of anti-CD3/28-based activation, the Treg cells secreted significantly more IFNγ, IL-4, IL-10, and other chemokines than controls (crucially no IL-2). From a functional perspective, the adoptive transfer of these cells into a RagKO murine colitis model found that the Treg cells lost FOXP3 expression after 8 weeks, there was an accumulation of IFNγ-producing Th1 cells and thus, the development of colitis was not prevented. Considering the data discussed in the previous paragraph, it is plausible that VHL KO-Treg cells were more susceptible to degradation of FOXP3 and the promotion of the effector Th1 program through binding to the HRE regions of the *IFN*γ gene. Overall, considering the data from this study of tTreg cells and studies from the above paragraphs of iTreg cells, it appears that HIF1α differentially modulates their differentiation, stability, and function. This raises additional challenges for understanding how Treg cells survive and function *in vivo* as environmental hypoxia would equally affect both cell subtypes.

### Metabolism

The effect of hypoxia on Treg cells’ metabolic pathways and how this influences cell function is not yet established (82). One would hypothesize that during hypoxia, Treg cells would adopt the non-oxygen-requiring glycolysis pathway to meet their metabolic demands. Indeed, the VHL KO Treg cells from the previous paragraph were found to have upregulated glycolysis-related genes. The glycolytic process also affected their function as the addition of 2-DG (glycolysis inhibitor) to these cells inhibited pro-inflammatory cytokine secretion. Collectively, this suggested that glycolysis was augmented during hypoxia, which in turn induced effector cell function in tTreg cells (81).

Regarding iTreg cells, the data suggest that hypoxia and glycolysis independently influence their metabolism (77). This was based upon several observations. Firstly, newly differentiated normoxic iTregs demonstrated a significantly lower rate of glycolysis than their newly differentiated Th1/2/17 counterparts. Secondly, the addition of 2-DG (glycolysis inhibitor) or rapamycin (mTOR inhibitor) to cultures of naïve CD4^+^ T-cells under Th17-differentiating conditions, prevented the adoption of the Th17-like phenotype. The cells still proliferated, however, they adopted a more iTreg-like phenotype instead - as demonstrated by the induction of FOXP3 and reduced secretion of IL-17A. Moreover, the 2-DG inhibited glycolysis directly and did not modulate glycolysis-related genes. Concerning hypoxia, the iTreg cells originating from a CD4-specific HIF1α-KO murine model expressed more FOXP3 and CTLA4 than their controls. Collectively, these data demonstrated that the induction of hypoxia and glycolysis were key to influencing CD4^+^ T-cell differentiation toward iTreg cells.

Overall, the roles of hypoxia in modulating Treg cell phenotype, function, and metabolism still need to be defined. We do not know if Treg cells still perform FAO despite hypoxia. We also do not know if by adopting glycolysis, whether they upregulate the PPP program for amino acid synthesis. Furthermore, a limitation of many studies is that they elucidated the effects of acute hypoxia only through *in vitro* experiments (Figure 3). In parallel, the KO models are unable to account for any tissue-specific effects of hypoxia and do not exclude the possibility of redundancy mechanisms to compensate for KO of HIF1/VHL. This issues are important for the liver allograft as its physiological hypoxia means that resident/circulating cells would have to adapt to survive/function in response to the chronically hypoxic microenvironment.

## Nuclear Receptors

The nuclear receptors are a unique family that interconnects lipophilic steroidal and non-steroidal ligands directly with DNA modulation (83) (Figure 1). A key difference in their mechanism is that steroidal receptors bind to DNA as homodimers whereas the non-steroidal receptors bind to DNA as heterodimers attached to the retinoid X receptor (RXR). In doing so, they collectively modulate genes responsible for cell differentiation, proliferation, function, and metabolism (83) (Figure 2). Whilst an in-depth discussion of these receptors is beyond the scope of this review, the ones implicated in promoting Treg cells include:

• Peroxisome proliferator-activator receptors (PPARα, β, γ).
• Liver X receptors (LXRα, β).
• Farsenoid X receptors (FXR).
• Vitamin D receptors (VDR).
• Retinoic acid receptor (RARα, β, γ).

### PPAR

The PPAR receptors are activated via fatty acids or pharmacological agonists (84). As previously discussed, acute activation of Treg cells upregulates mTORC1 activity, increases glycolysis and fatty acid catabolism. However, recent work in CD4^+^ T-cells has demonstrated that the mechanism for fatty acid catabolism is dependent on mTORC1 inducing PPARγ as well as the sterol regulatory element-binding protein 1 (SREBP1) (84). The PPARγ gene promoted the expression of genes to take-up lipids, perform fatty acid synthesis, and lipolysis too. Moreover, the induction of PPARγ was also important in promoting the proliferation and activation of CD4^+^ T-cells (84).

The role of PPARγ in Treg cells has been particularly investigated in visceral adipose tissue (VAT). For example, the expression of both PPARγ and FOXP3 was identified as crucial to inducing the genetic signature of VAT-specific Treg cells (85). PPARy agonism via thiazolidinediones upregulated genes crucial to both Treg cell metabolism and function. For example, genes for lcFA metabolism such as CD36, CPT1 as well as fatty acid synthesis were upregulated. In parallel, there was also an increase in the expression of FOXP3 and Gata3. Most importantly, these changes were identified in VAT-resident Tregs only, which suggested a role for the lipid-rich microenvironment in local immunoregulation.

Similar effects were also identified in the tumor setting via the PPARβ receptor (45). The CD36-based triggering activated the PPARβ signaling pathway which promoted lipid metabolism and function in Treg cells. This process involved increased FOXP3 expression, mitochondrial function, and the NAD/NADH ratio. Most notably, in this tumoral setting PPARγ was not identified as being affected via CD36 signaling. This suggests that the PPAR isoforms could be differentially implicated in Treg cell metabolism/function depending on the tissue setting.

### LXR

There are two forms of the LXR receptor (α, β) of which LXRβ is universally present on all tissues whereas LXRα is specific to certain tissues e.g., hepatic, adipose and gut (86, 87). LXRs are activated by oxidized cholesterol derivatives (oxysterols) and propagate a genetic response that involves increased cholesterol and lipid metabolism (86). In particular, they augment the expression of ABC-cassette transporters which are responsible for the excretion of sterols (88).

Concerning Treg cells, there is *in vitro* data demonstrating that culturing murine CD4^+^ T-cells under iTreg-differentiating conditions with LXR agonists significantly increased the expression of FOXP3, reduced IFN-γ, and IL-17A secretion (89). These iTreg cells were also more immunosuppression during *in vitro* assays. This effect was confirmed *in vivo* with oral LXR agonists as an increase in intestinal accumulation of Treg cells was identified.

LXR agonists are presently undergoing clinical trials as anti-inflammatory agents in atherosclerosis, however, an increase in hepatic steatosis has been identified as a key side effect. From this perspective, another group developed an LXR inverse agonist which demonstrated a reduction in murine models of hepatic steatosis instead (90). Put together, these data demonstrate that we still need to further understand the implications of LXR-mediated DNA modulation before progressing to clinical trials.

### FXR

The FXR receptors are particularly pertinent in liver physiology as their key ligands are biliary acids. Upon activation, FXR receptors induce the transcription of genes specific for transporters that facilitate biliary efflux and inhibit genes responsible for biliary acid synthesis (91). However, although the FXR receptor is highly expressed in hepatic tissue, very low levels of FXR mRNA have been identified in all peripheral blood mononuclear cells e.g., T/B-cells, monocytes) (92). Hence, it is likely that if biliary acids do impact Treg cell function, this occurs indirectly. The two potential mechanisms could be (1) FXR-independent modulation of Treg cells or (2) FXR modulation of non-Treg cells whose downstream effects involve Treg cells. Indeed, evidence for both such mechanisms has been recently reported.

(1) The isoallolithocholic (isoallo-LCA) biliary acid was shown to modulate Treg cells independently of FXR (93). In cultures of murine CD4^+^ T-cells under iTreg-differentiating conditions with isoallo-LCA, the expression of FOXP3 was significantly increased compared to other biliary acids. The mechanism of action of isoallo-LCA involved interacting with the conserved nuclear sequence 3 (CNS3) on the FOXP3 gene to indirectly promote FOXP3 acetylation. Moreover, these iTregs were able to suppress colitis development upon adoptive transfer – thus demonstrating superior *in vivo* immunosuppressive capacity. From a metabolic perspective, isoallo-LCA increased the OCR and superoxide levels in iTregs. Put together, this work demonstrated how isoallo-LCA could both augment mitochondrial-based metabolism and promote iTreg cell function.
(2) In comparison, the omega-muricholic and 3β-hydroxydeoxycholic (isoDCA) acids were found to significantly increase FOXP3 expression on naïve murine CD4^+^ T-cells when co-cultured with DCs (94). This effect was not possible when the biliary acids were cultured with naïve CD4 T-cells only – thus, indicating an indirect DC-based effect. Indeed, the mechanism involved isoDCA having an antagonistic effect upon binding to the FXR receptor on DCs and downregulating a range of pro-inflammatory genes. Through a range of innovative murine models and engineered microbes, they confirmed that these effects by demonstrating how these biliary acids could induce colonic pTreg cells. However, the exact nature of the interaction between the DCs and naïve CD4^+^ T-cells was not identified. Moreover, the effects of these biliary acids on the metabolism of the iTreg/pTreg cells were not explored either.

### VDR

The VDR receptor in T-cells is activated by the active form of vitamin D, 1,25-dihydroxyvitamin D3 (83). The receptor then forms a heterodimeric complex with RXR, which can bind to specific sections of DNA (called vitamin D response elements; VDRE) (95). This propagates the transcription of a range of protein complexes responsible for enacting T-cell functions.

The addition of active vitamin D into cultures of activated human CD4^+^ T-cells or CD4^+^CD25^–^ T-cells has been shown to significantly increase FOXP3 expression compared to controls (96, 97). In parallel, it also increased expression of CTLA4 and reduced secretion of IFN-γ, IL-17A, and IL-2. These iTregs were better able to suppress the proliferation of Teff cells during *in vitro* assays and they secreted slightly more IL-10 too. However, these iTregs were unstable as FOXP3 expression declined after day 4 of activation. In a different study, VDR was also able to induce functioning iTregs from Th2 cells (98). Overall, although VDR activation can induce Treg cells, it is unknown how (and if) VDR in parallel modulates Treg cell metabolism.

### RAR

The RAR group of receptors in T-cells are activated by the active form of vitamin A, all-trans retinoic acid (ATRA) (83). Similar to VDR, they form a heterodimeric complex with RXR, which then binds to the complementary sections of DNA (called retinoic acid response elements; RARE).

With respect to tTreg cells, *in vitro* cultures of ATRA and rapamycin with activated human Treg cells (defined as CD4^+^CD25^+^CD127^lo^ in the study) found that the combined ATRA/rapamycin group significantly increased FOXP3 expression on their Treg cells. These cells also demonstrated superior immunosuppressive capacity (99). Similar effects were also identified in experiments investigating ATRA in TGF-β-induced Tregs (100). However, it is unknown how (if at all) ATRA modulates Treg cell metabolism.

## Translational Potential of Treg Cell Metabolism

The aim of targeting Treg cell metabolism for cellular therapy applications is to induce specific metabolic pathways that augment cellular survival/function (Figure 1). These optimized Treg cells could outcompete Teffs metabolically in the allograft and thus, inhibit their survival and suppress their effector activity (34). Treg cells also have the potential for Th17 plasticity so combined immunometabolic optimization could prevent Treg cell interconversion in an inflammatory allograft (32, 101). A further advantage of optimizing Treg cell activity is to exploit their bystander suppressor functions and thereby reduce the effector functions of other pro-inflammatory cells (102).

**FIGURE 4:**
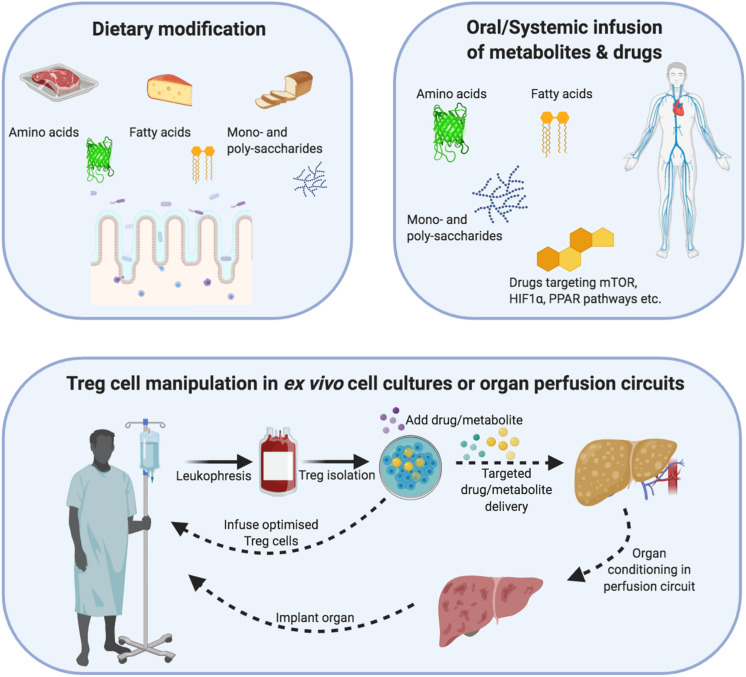
Demonstrating how regulatory T-cells can be metabolically optimized through dietary changes, *ex vivo* cellular therapy or organ reperfusion machines. **Dietary modification/supplementation:** Different metabolites differentially modulate Treg cell metabolism and function. Treg cells metabolize mainly via non-mitochondrial (glycolysis) and mitochondrial e.g., oxidative phosphorylation and fatty acid oxidation) means. In addition, their internal cofactors such as mTOR, PPAR and HIF1α allow Treg cells to respond to substrate/oxygen changes in the microenvironment. Dietary modification or systemic infusions of drugs/metabolites could be used to augment Treg cell metabolism and function in an inflammatory allograft. **Cellular therapy:** Cell therapy involves the isolation of autologous Treg cells, which undergo expansion under sterile Good Manufacturing Principle (GMP) conditions. During this expansion process, the function and metabolism of Treg cells can be modified through drugs or change the substrate composition of metabolites. After the expansion process, the Treg cells could be returned to the patient or infused into an organ reperfusion circuit. **Organ reperfusion:** The recent introduction of organ reperfusion machines into clinical practice is changing the *modus operandi* of transplantation. Extended-criteria organs can be re-conditioned using different oxygen-delivery molecules, cytokines, drugs and metabolites to reduce their intrinsic ischemia/inflammatory burden as well as support the function of resident Treg cells. In parallel, recipient-expanded Treg cells could be infused during the reperfusion process to further optimize the reconditioning process. The recovery of an organ can then be measured through objective criteria e.g., pH, bile acid, lactate) and a decision made to implant or discard the organ.

This combined immunometabolic modulation could be performed either *in vivo* or *ex vivo* (Figure 4). Concerning the *in vivo* approach, this would involve either dietary supplementation of substrates e.g., fatty acids, amino acids) or systemic administration of substrates/drugs. This approach is clinically feasible and could be periodically delivered in response to post-transplant protocol biopsies. However, to ensure its efficacy, there will need to be additional pharmacokinetic studies and even utilization of novel drug delivery approaches for Treg-specific targeting. In comparison, the *ex vivo* approach would involve culture media supplementation with the relevant substrates/drugs during the GMP process. In this way, the Treg cells could be specifically targeted and the substrates/drugs leftover could be washed out at the end of the culture process.

### Metabolite Supplementation

Treg cells utilize glycolysis, FAO, and OXPHOS as their constitutive metabolic programs (Figure 2). During activation, Treg (and Teff) cells upregulate the expression of Glut1 transporters and their rate of glycolysis (ECAR). Hence, to allow Treg cells to outcompete their effector counterparts, supporting FAO could be a viable therapeutic approach instead. This would involve either direct supplementation of fatty acids, or inhibiting FASyn (via C75, soraphen) (26, 43, 44). In the past, direct supplementation with scFAs has been attempted, however, perhaps using lcFAs or polyunsaturated FAs would be more efficient in terms of ATP generation (26) (Figure 2). An opposite approach would involve targeting Teff cells by inhibiting glucose uptake (via Glut transporters), glycolysis e.g., 2-DG), or inhibiting fatty acid uptake (CD36), fatty acid transport (a) or mitochondrial uptake (CPT1) (60, 103–106).

However, we do not know yet which metabolic pathways are utilized or favorable to a graft experiencing acute or chronic rejection. It is also not known whether over usage of particular metabolic pathways would increase oxidative stress and propagate early apoptosis (39) (Figure 2). From a pharmaceutical perspective, although systemic infusion of dietary metabolites would be a route of administration, one could also utilize the oral route for improved bioavailability in intestinal and hepatic allografts (107). However, further pharmacokinetic studies are needed to assess these approaches in-depth.

Finally, a novel point of intervention could be through *ex vivo* organ machine perfusion technologies (108–110) (Figure 4). These are increasingly being used for conditioning to improve organ quality and subsequent patient outcomes. Upon explantation, the organ is first connected to a circulatory circuit within the perfusion machine. During the hours that follow (depending on the platform), the organ physiology can be monitored using a range of quality-control criteria such as color, pH, lactate levels, and even bile production. This period of perfusion would be optimal for metabolic-based immunomodulation.

### Pharmacological Modulation

In this manuscript, we have discussed the internal immunometabolic axes of Treg cells that could be modulated to optimize both metabolic/functional pathways.

#### mTOR

Mammalian transporter of rapamycin inhibition via rapamycin is a part of current immunosuppression protocols as well as *ex vivo* GMP Treg cell expansion protocols (1, 19, 20, 111). This is because rapamycin improves Treg cell expansion, FOXP3 expression, and immunosuppressive function (25). Another way of inhibiting mTOR involves metformin, which is an AMPK kinase activator (73, 112). In Treg cells, it can inhibit FASyn by targeting ACC (112). This could augment FAO activity to outcompete the Teff cells.

However, the mTOR inhibitory approach is complicated by data discussed in previous sections demonstrating divergent roles of mTOR signaling in Treg cells (14, 53) (Figure 2). mTOR signaling improves Treg cell proliferation, glycolysis, lipid metabolism, and OXPHOS. Furthermore, essential amino acids also promote mTORC1 activity in Treg cells. Indeed, the ongoing presence of amino acids is necessary for Treg cells to sustain mTORC1 activity and Treg cell function (15, 68). To reconcile the differences in published studies, although the idea of an mTOR “oscillatory switch” has been hypothesized, it does mean that any mTOR-based modulation needs to be carefully refined for clinical benefit (113).

#### Hypoxia

As discussed in the Hypoxia section, the overall effects of hypoxia signaling in Treg cells are unclear. In tTreg cells, HIF1α seems to reduce (or not affect) FOXP3 expression, augments glycolysis, and induce effector activity. In comparison, HIF1α induces FOXP3 in iTregs (Figure 2).

In terms of manipulating tTreg cells during cell therapy or targeting allograft resident tTregs, increasing either local oxygen delivery or reducing HIF1α levels could be therapeutic approaches (Figure 4). Indeed, non-blood-based oxygen carriers have already been investigated in normothermic machine perfusion of the liver (114). The livers were more efficient at taking up the stored oxygen from these carrier molecules than hemoglobin. With regard to HIF1α levels, a range of HIF1α-targeting agonists/antagonists are either in trials or clinically available (115–117). These could either be systemically administered to patients or utilized in GMP culture protocols or machine perfusion technologies.

#### Nuclear Receptors

Nuclear receptor agonism could more precisely target Treg cell metabolism than oxygen-based or mTOR modulation. For example, PPARβ/γ agonism has been shown to jointly promote FOXP3 expression, FAO, FASyn, and mitochondrial function in Treg cells. Indeed, PPARγ agonists such as thiazolidinediones are clinically available and have been used for many years in diabetes already (118). Many patients with kidney allografts will be used to taking them too.

In comparison, although LXR agonists are being studied pre-experimentally in the settings of atherosclerosis/dyslipidemia, their noted side effect of inducing hepatic steatosis is a safety concern (119). Concerning the FXR receptor, indirect modulation via biliary acids is likely to be more challenging. There will need to be a range of pharmacokinetic studies to study their composition as well as intestinal/hepatic bioavailability. Furthermore, ensuring the specificity of their action on Treg cells only will require the use of more novel drug delivery approaches. Hence, LXR and FXR modulation is not a currently feasible Treg cell modulation strategy in transplantation.

## Future Challenges

In summary, this review has outlined numerous ways in which Treg cell metabolism could be exploited for therapeutic benefit in transplantation. Through our discussions, we have also highlighted crucial ongoing knowledge gaps. The following themes need to be addressed in detail if we are going to move forward with translational Treg cell immunometabolism:

• When should Treg cells be administered post-transplant?
• Do antigen-specific or genetically engineered Treg cells metabolize differently?
• How can drugs and metabolites be delivered specifically to *in vivo* Tregs cells?
• At which time point post-infusion will Treg-boosting metabolites need to be administered?
• Can *in vivo* Treg cell metabolic programs be switched on/off as per clinical need?
• How can Treg cell metabolic activity be monitored in the graft?

Although the focus of this review has been on Treg cell metabolism, there is also a global cohort of teams actively researching other novel approaches such as genetic engineering, improved donor antigen-specificity, and epigenetics to optimize Treg cells. In light of the acceptable safety profile of Treg cells demonstrated in recent clinical trials, we can look forward to these novel approaches being exploited to optimize Treg cell efficacy. The coming years are indeed going to be exciting for Treg cell therapy – both for us and for our patients.

## Author Contributions

MA and MM designed, wrote, and revised the manuscript. AM, FC, OS, and GG advised on the editing and helped to revise the manuscript. All authors contributed to the article and approved the submitted version.

## Conflict of Interest

The authors declare that the research was conducted in the absence of any commercial or financial relationships that could be construed as a potential conflict of interest.
